# Pressure‐Driven Phase Transition Unlocking Unique Eu^2+^ Luminescence in Li_2_SrSiO_4_ for Optical Sensing and White‐LEDs

**DOI:** 10.1002/advs.75887

**Published:** 2026-06-02

**Authors:** Przemysław Woźny, Peng Du, Teng Zheng, Junpeng Xue, Liang Peng, Shuailing Ma, Szymon Sobczak, Victor Lavín, Tian Cui, Marcin Runowski

**Affiliations:** ^1^ School of Information and Electrical Engineering Hangzhou City University Hangzhou Zhejiang China; ^2^ Faculty of Chemistry Adam Mickiewicz University Uniwersytetu Poznańskiego 8 Poznań Poland; ^3^ School of Physical Science and Technology Ningbo University Ningbo Zhejiang China; ^4^ School of Science Jiangsu University of Science and Technology Zhenjiang China; ^5^ Departamento de Física MALTA‐Consolider Team & IUdEA Universidad De La Laguna Apartado de Correos 456 San Cristóbal de La Laguna Santa Cruz de Tenerife Spain

**Keywords:** diamond anvil cell, Eu^2+^ ions, high‐pressure synthesis, optical manometry, remote pressure sensors

## Abstract

Controlling the luminescence behavior of rare‐earth‐doped materials under extreme physical conditions of pressure and/or temperature is crucial for the development of next‐generation solid‐state lighting and optical sensors. Here, the Li_2_SrSiO_4_:3%Eu^2+^,0.5%Cs^+^ phosphors were synthesized and permanently modified by high‐pressure treatment to design high‐performance white‐LED and optical manometer. With Cs^+^ introduction, the luminescence intensity, quantum efficiency, and thermal stability of the phosphors increased. Using the designed phosphor as a yellow‐emitting component, a white‐LED was fabricated. The studied material was also used to develop a multiparametric optical pressure and temperature sensor. Importantly, compression above 14.0 GPa induced formation of a new high‐pressure β‐phase, which can be recovered under ambient conditions, and ‐ exhibits an unique pressure‐induced emission band. Photoluminescence spectroscopy revealed the presence of yellow and a new blue emission component in the emission spectrum, providing sensitivities of the synthesized phosphors over 9 and 5 nm GPa^−1^, respectively. Moreover, the application of high pressure in a large‐format press (up to 20 GPa) enabled the fabrication of a white‐LED device with improved color performance. Such large‐scale, high‐pressure postsynthetic treatment for the development of sensors and new light sources is reported here for the first time.

## Graphical abstract

This study demonstrates, for the first time, the use of a large‐format high‐pressure press to modify synthesized Li_2_SrSiO_4_:Eu^2+^,Cs^+^ phosphors after its preparation. The pressure treatment results in the formation of new materials with distinctive optical properties, opening pathways for the development of advanced LED devices and high‐performance optical manometers.

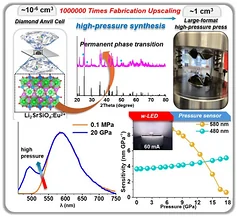

## 1 Introduction

Inorganic materials with the general formula A_2_BCO_4_ (where A = alkali metal; B = metal, C = semimetal), exhibit unique, ordered cation arrangements that enable tunable electronic and optical properties [1, 2, 3]. Among these, silicate‐based materials (A_2_BSiO_4_) have gained increasing interest as promising alternatives to the simpler crystal framework of ABX_3_ perovskite [4, 5, 6, 7, 8, 9]. In this regard, the more complex structure offers greater flexibility in terms of chemical composition and physical functionality in advanced applications. Their structural versatility, defect tolerance, and ordered B/C sublattice enable bandgap and carrier dynamics engineering, enhancing photoluminescence (PL) and making them ideal for energy‐efficient optoelectronic devices, i.e. photovoltaics, solar concentrators, LEDs, photodetectors, etc [10, 11, 12, 13, 14, 15, 16, 17]. Doping materials with optically active lanthanide ions (e.g. Eu^2+^_,_ Sm^2+^, Ce^3+^, Yb^3+^), which possess characteristic PL properties, enables modification of the luminescence properties of the host matrix and holds promise for next‐generation luminescent material applications [18, 19, 20, 21, 22]. For instance, the divalent Eu^2+^ ions show a broad emission band due to *4f^6^5d^1^→4f^7^* transitions, which is highly sensitive to the crystal field strength, enabling detection of local symmetry changes under compression by a shift in the spectral position of the emission band [23, 24, 25, 26].

Lithium silicate materials with the general formula Li_2_BSiO_4_, including Li_2_SrSiO_4_, Li_2_EuSiO_4_, Li_2_CaSiO_4_, Li_2_BaSiO_4_, Li_2_MgSiO_4,_ Li_2_CoSiO_4_ and other silicates have been widely studied for applications in light‐emitting diode, Li‐ion batteries, field emission display, optical thermometry, etc [27, 28, 29, 30, 31, 32, 33, 34, 35, 36]. Li_2_SrSiO_4_ crystallizes adopting both hexagonal or triclinic structures and is widely applied due to its superior PL properties, low cost, good thermal and chemical stability. The structure of Li_2_SrSiO_4_ consists of layers of ions in a hexagonal close‐packed arrangement. Cations occupy the octahedral and tetrahedral sites, with tetrahedral units of SiO_4_ and LiO_4_ forming a three‐dimensional network. The Sr^2+^ ions, surrounded by eight O atoms, are located in the interstitial spaces outside the network. Three‐eighths of the tetrahedral sites are occupied by Li^+^ and Sr^2+^ ions, while Si^4+^ occupies one‐eighth of the sites. Furthermore, many studies have shown that co‐doping with Cs^+^ in optical materials leads to more intense luminescence, due to the stabilization of Li vacancies, which suppresses the oxidation of Eu^2+^ ion [29, 32]. However, the studies about the impact of co‐doping Cs^+^ in Li_2_SrSiO_4_:3%Eu^2+^ are still lacking. The excitation and emission spectra of Eu^2+^‐doped phosphors can be tuned in different hosts and/or crystallographic sites due to effects such as the nephelauxetic effect, crystal splitting effect, and Stokes shift [1, 37, 38].

Pressure and temperature, as fundamental physical parameters, have a huge impact on material properties [39]. Luminescence features are especially sensitive to material structure modifications under high pressure, making them ideal for monitoring pressure‐induced changes. In situ spectroscopy under extreme conditions can reveal phase transitions and discover novel characteristics in the compressed materials and newly formed high‐pressure structures [40, 41, 42]. Such studies provide indirect insights into elastic (reversible) and plastic (irreversible) deformations, altered atomic arrangements, electronic configurations and interatomic distances often leading to the formation of novel, unique materials with tailored optical and electronic properties [43, 44, 45]. Such studies are crucial for aerospace, electronics, and energy sectors, where high‐performance materials are needed. Pressure can induce coordination‐environment changes around rare earth ions, directly impacting their luminescence [46]. These changes modulate their electronic states, leading to shifts of emission bands, intensity change, and luminescence lifetime modifications [12, 47, 48]. Reversible pressure‐induced changes are exploited in optical manometers for precise pressure sensing, while irreversible transformations result in discovering new, unique material phases with distinct properties [10, 46, 49, 50, 51, 52, 53, 54]. Materials compression in the diamond anvil cell (DAC) allows the study of structural changes, electronic transitions, and optical properties due to the high transparency of diamonds for electromagnetic radiation [55, 56, 57]. Growing interest in this type of research is driven by the discovery of unique phenomena, such as superconductivity, magnetism, amorphization, and pressure‐tuned luminescence [58, 59, 60, 61, 62, 63]. Large‐format high‐pressure presses complement DAC for larger‐scale synthesis, enabling the bulk, scaled‐up production of pressure‐tailored materials [64, 65, 66].

Here, we have investigated the Li_2_SrSiO_4_:3%Eu^2+^,0.5%Cs^+^ materials with optimized content of Eu^2+^ ions to maximize luminescence intensity, and Cs^+^ ions providing stabilization of the resulting structure, leading to the overall enhancement of luminescence while reducing the thermal quenching. We investigate the Cs^+^ dopant influence on the structural and luminescence properties of the Li_2_SrSiO_4_:3%Eu^2+^ materials. Temperature‐dependent luminescence and thermal sensing properties of undoped and Cs^+^‐doped materials were also compared. Moreover, properties (i.e., absorption, emission, Raman spectra, and XRD patterns) of the optimized Li_2_SrSiO_4_:3%Eu^2+^,0.5%Cs^+^ were investigated in high‐pressure conditions. Finally, a new high‐pressure phase of the triclinic β‐Li_2_SrSiO_4_:3%Eu^2+^,0.5%Cs^+^ was obtained, which is stable at ambient conditions and exhibits unique photoluminescence properties.

## 2 Results and Discussion

### 2.1 Properties at Ambient Conditions

**FIGURE 1:**
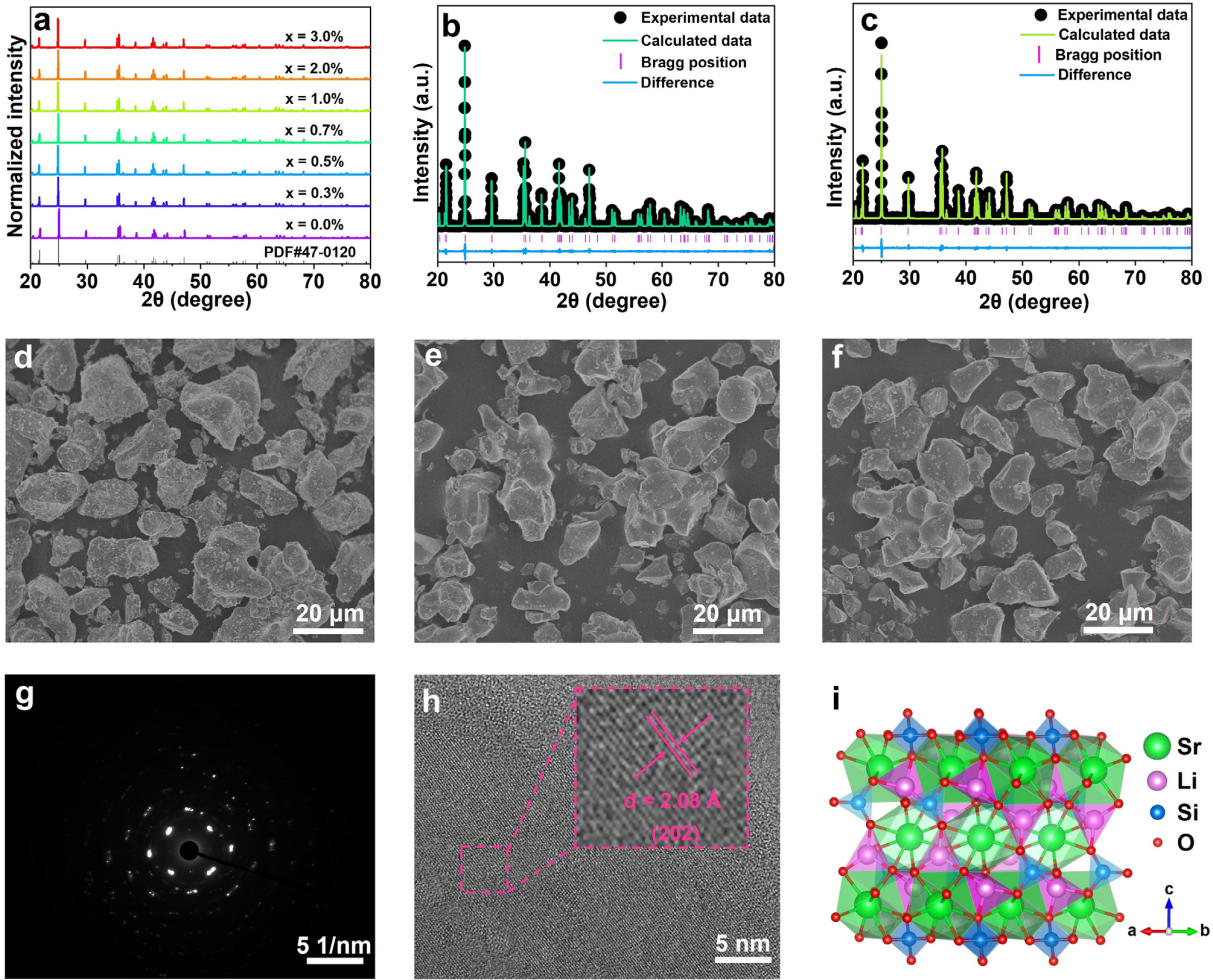
(a) Powder XRD patterns of the Li_2_SrSiO_4_:3%Eu^2+^,*x*Cs^+^ phosphors. Rietveld XRD patterns of the (b) Li_2_SrSiO_4_:3%Eu^2+^ and (c) Li_2_SrSiO_4_:3%Eu^2+^,0.5%Cs^+^ phosphors. SEM images of the Li_2_SrSiO_4_:3%Eu^2+^,*x*Cs^+^ phosphors with the *x* value of (d) 0.0%, (e) 0.5%, and (f) 3%. (g) SAED pattern, (h) High‐resolution TEM image, and (i) 3D representation (unit cell) of the Li_2_SrSiO_4_ crystal structure.

The X‐ray diffraction patterns of the Li_2_SrSiO_4_:3%Eu^2+^,*x*Cs^+^ phosphors were examined to explore their phase compositions caused by Cs^+^ ions doping. Zhou et al. (2020) reported that the optimal Eu^2+^ ion concentration in a similar phosphor material was 3%, yielding improved luminescence efficiency. Accordingly, we employed this concentration in our study [28]. As displayed in Figure 1a, all the materials show identical diffraction reflexes, and they are in good accordance with the standard hexagonal Li_2_EuSiO_4_ (PDF#47‐0120), isostructural with Li_2_SrSiO_4_, confirming that the final products have a pure phase, which is scarcely impacted by Eu^2+^ and Cs^+^ doping. To get deep insight into the phase structure of the final compounds, the Rietveld XRD refinements of the representative Li_2_SrSiO_4_:3%Eu^2+^ and Li_2_SrSiO_4_:3%Eu^2+^,0.5%Cs^+^ phosphors were carried out, and the corresponding results are shown in Figure 1b,c, respectively. Evidently, the calculated diffraction profiles match well with the experimental data, demonstrating the single phase of the obtained phosphors. Moreover, as Cs^+^ ions are introduced, the lattice parameters of the studied samples are slightly increased (see Table S1), which is assigned to the smaller Li^+^ is replaced by the larger Cs^+^.

The morphological features of the designed phosphors were characterized via SEM and TEM techniques. From the recorded SEM images, it is clear that the resulting phosphors are composed of irregular micron‐sized particles, whose size and morphology are independent of the Cs^+^ content, as shown in Figure 1d–f. The mean particle sizes of Li_2_SrSiO_4_:3%Eu^2+^,*x*Cs^+^ (*x* = 0, 0.5, 3%) obtained via grinding were estimated to be 6.20, 5.65, and 5.56 µm, respectively. The detailed distributions are shown in the histograms in Figure S1a–c. Furthermore, it can be seen that the selected area electron diffraction (SAED) pattern contains many bright dots (Figure 1g), showing the single‐crystal nature of the final material. As presented in Figure 1h, the high‐resolution TEM image reveals distinct lattice fringes with interplanar distance of 2.08 Å, corresponding to the (202) plane of Li_2_SrSiO_4_. In Figure 1i the 3D visualization of the Li_2_SrSiO_4_ crystal structure is presented.

**FIGURE 2:**
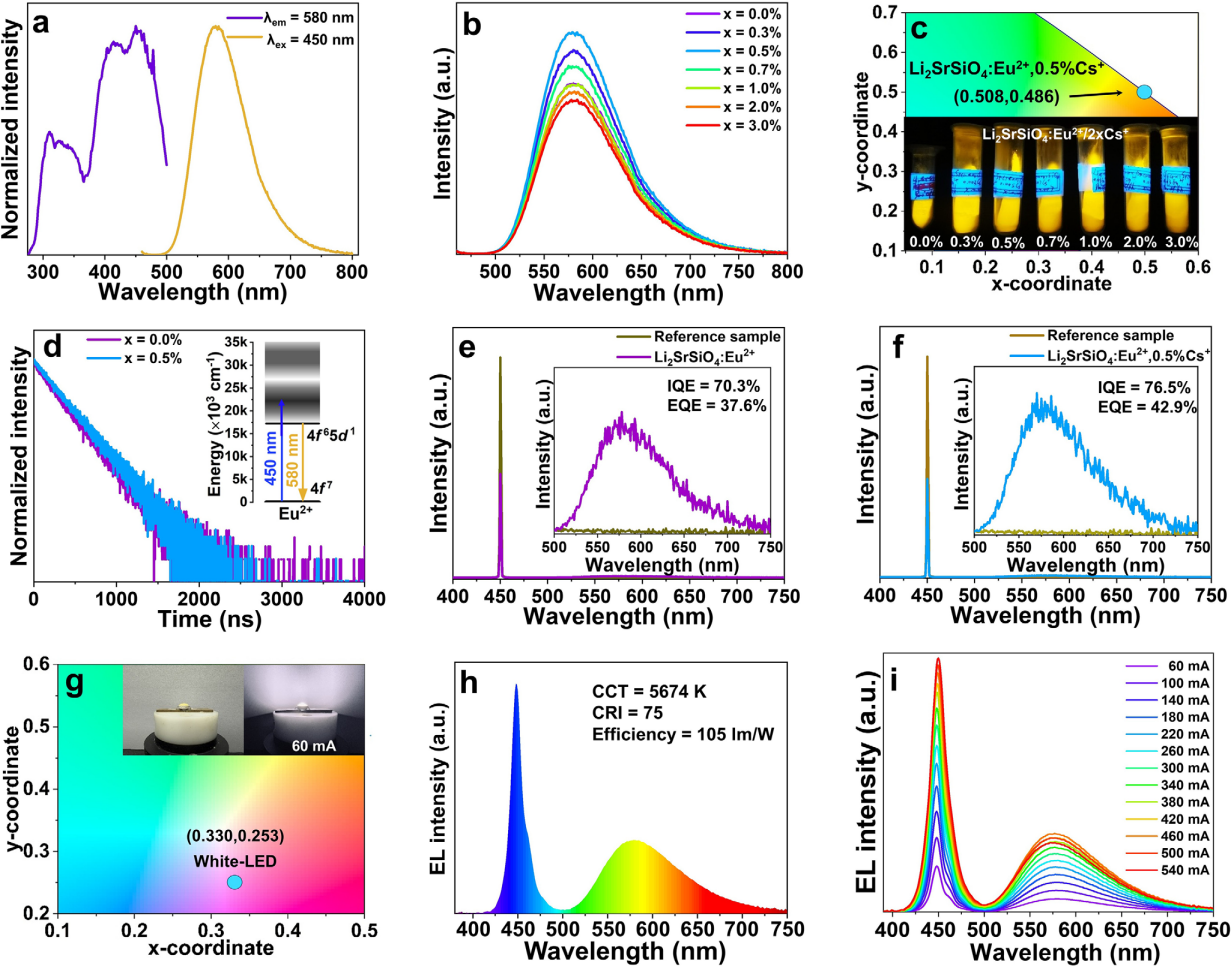
(a) Excitation and emission spectra of the Li_2_SrSiO_4_:3%Eu^2+^,0.5%Cs^+^ phosphors. (b) Emission spectra of the Li_2_SrSiO_4_:3%Eu^2+^,*x*Cs^+^ phosphors. (c) CIE chromaticity diagram of the Li_2_SrSiO_4_:3%Eu^2+^,0.5%Cs^+^ phosphors. The inset shows the optical images of the resulting phosphors irradiated by NUV light. (d) Luminescence decay curves of the Li_2_SrSiO_4_:3%Eu^2+^ and Li_2_SrSiO_4_:Eu^2+^,0.5%Cs^+^ phosphors. Quantum efficiency measurements of the (e) Li_2_SrSiO_4_:3%Eu^2+^ and (f) Li_2_SrSiO_4_:3%Eu^2+^,0.5%Cs^+^ phosphors. (g) CIE chromaticity diagram of the packaged white‐LED recorded at 60 mA; the inset depicts the optical images of the fabricated device. (h) EL emission spectra of the packaged white‐LED recorded at 60 mA. (i) EL spectra of the designed white‐LED as a function of working current in the range of 60–540 mA.

To explore the impact of Cs^+^ doping on the luminescence properties of the studied materials, their excitation and emission spectra were recorded. When monitored at 580 nm, the studied samples show two strong, non‐symmetric excitation bands in both UV and blue regions characteristic for Eu^2+^ ions, which arise from the interconfigurational 4*f*‐5*d* transition of Eu^2+^ ions, as shown in Figure 2a [67]. Excited at 450 nm, the emission spectrum has an intense broad band at 580 nm, corresponding to the electronic transition from 5*d* to 4*f* level, i.e., *4f^6^5d^1^→4f^7^* (Figure 2a) [28, 68]. Interestingly, according to the excitation wavelength‐dependent emission spectra (Figure S2a), it is clear that the resultant phosphors can be efficiently excited by both NUV and blue radiation, while the emission band position and shape are independent of the excitation wavelength, which is beneficial for applications in solid‐state lighting. Moreover, for the purpose of exploring the optimal doping content of Cs^+^ ions in the studied samples, the emission spectra of the Li_2_SrSiO_4_:3%Eu^2+^,*x*Cs^+^ phosphors were recorded and presented in Figure 2b. As Cs^+^ content increases up to *x* = 0.5%, it can be seen that the luminescence intensity increases. Since the ionic radius of Cs^+^ is much larger than that of Li^+^, the lattice expansion will take place when Li^+^ is replaced by Cs^+^, resulting in the modified crystal field surrounding of the Eu^2+^ in the Li_2_SrSiO_4_ host lattice. Thereby, the enhanced luminescence properties are observed in the designed phosphors. Note that the concentration quenching effect also occurs when *x* > 0.5%. When the resulting phosphors are irradiated by NUV radiation, they are capable of emitting bright yellow light, with the color coordinates of the Li_2_SrSiO_4_:3%Eu^2+^,0.5%Cs^+^ phosphors being (0.508, 0.486), as displayed in Figure 2c. The representative decay curves of the Li_2_SrSiO_4_:3%Eu^2+^ and Li_2_SrSiO_4_:3%Eu^2+^,0.5%Cs^+^ phosphors are shown in Figure 2d. Evidently, the recorded decay profiles can be fitted by a single exponential function, as described by Equation (1) below:

(1) $$I\;\left(t\right)=I_{0}+A\exp\left(-t/\tau \right)$$

where *I(t)* and *I_0_* are the luminescence intensities at time *t* and *t* = 0, respectively, *A* is a constant (amplitude) and *τ* is the decay time. Accordingly, the excited state lifetimes of the Li_2_SrSiO_4_:3%Eu^2+^ and Li_2_SrSiO_4_:3%Eu^2+^,0.5%Cs^+^ phosphors are found to be 285 ± 5 and 310 ± 5 ns, respectively. Furthermore, the luminescence process of the studied materials is described by the energy level diagram of Eu^2+^, as shown in the inset of Figure 2d. In terms of the practical applications, the studied phosphors are expected to exhibit high quantum efficiency. Herein, excited at 450 nm, the quantum efficiencies of the Li_2_SrSiO_4_:3%Eu^2+^ and Li_2_SrSiO_4_:3%Eu^2+^,0.5%Cs^+^ phosphors were measured (see Figure 2e,f). The following expressions (Equations (2), (3), (4)) were used to estimate the internal (IQE) and external (EQE) quantum efficiencies of the studied samples: [69]

(2) $$IQE=\int L_{S}/\left(\int E_{R}-\int E_{S}\right)$$

(3) $$AE=\left(\int E_{R}-\int E_{S}\right)/\int E_{R}\;$$

(4) EQE=AE×IQE

where the excitation line without and with the studied samples are labeled by *E_S_* and *E_R_*, respectively. It is found that *IQE* and *EQE* values of the Li_2_SrSiO_4_:3%Eu^2+^,0.5%Cs^+^ phosphors are 76.5% and 42.9%, respectively, which are higher than those of the Li_2_SrSiO_4_:3%Eu^2+^ phosphors (i.e., *IQE* = 70.3% and EQE = 37.6%), further confirming that the introducing of Cs^+^ is an efficient route to enhance the luminescence properties of the studied Li_2_SrSiO_4_:3%Eu^2+^ material. These results demonstrate that the designed phosphors show very good luminescence properties, allowing their usage in optoelectronic applications.

In an attempt to explore the feasibility of the designed phosphors in solid‐state lighting applications, a white‐LED was prepared by using the Li_2_SrSiO_4_:3%Eu^2+^,0.5%Cs^+^ phosphors as the yellow‐emitting converter, by combining it with a commercial blue chip (Figure 2g). When the working current is 60 mA, it can be seen that the developed white‐LED can emit bright white light and its chromaticity coordinates, correlated color temperature (CCT), color rendering index (CRI) and luminous efficiency are (0.330, 0.253), 5674 K, 75, and 105 lm/W, respectively (see Figure 2h). Figure 2i shows the emission spectra of the fabricated white‐LED, where only the emission bands of the blue chip and optimized phosphors are observed. Interestingly, the emission intensity of the developed white‐LED increases gradually with increasing driving current in the range of 60–460 mA, reaching a maximum at 460 mA. Above this current, a saturation effect is observed, and the luminescence intensity starts to decrease, with saturation occurring above 460 mA. For comparison, the commercially used YAG:Ce^3+^ phosphor was also measured, showing saturation above 580 mA (Figure S2b). Moreover, it is found that the emission characteristics of the fabricated white‐LED are also slightly impacted by the driving current, as listed in Table S2. These results directly reveal that the synthesized phosphors can be used for high‐power solid‐state lighting as a yellow‐emitting component.

### 2.2 Temperature‐Dependent Photoluminescence Properties

**FIGURE 3:**
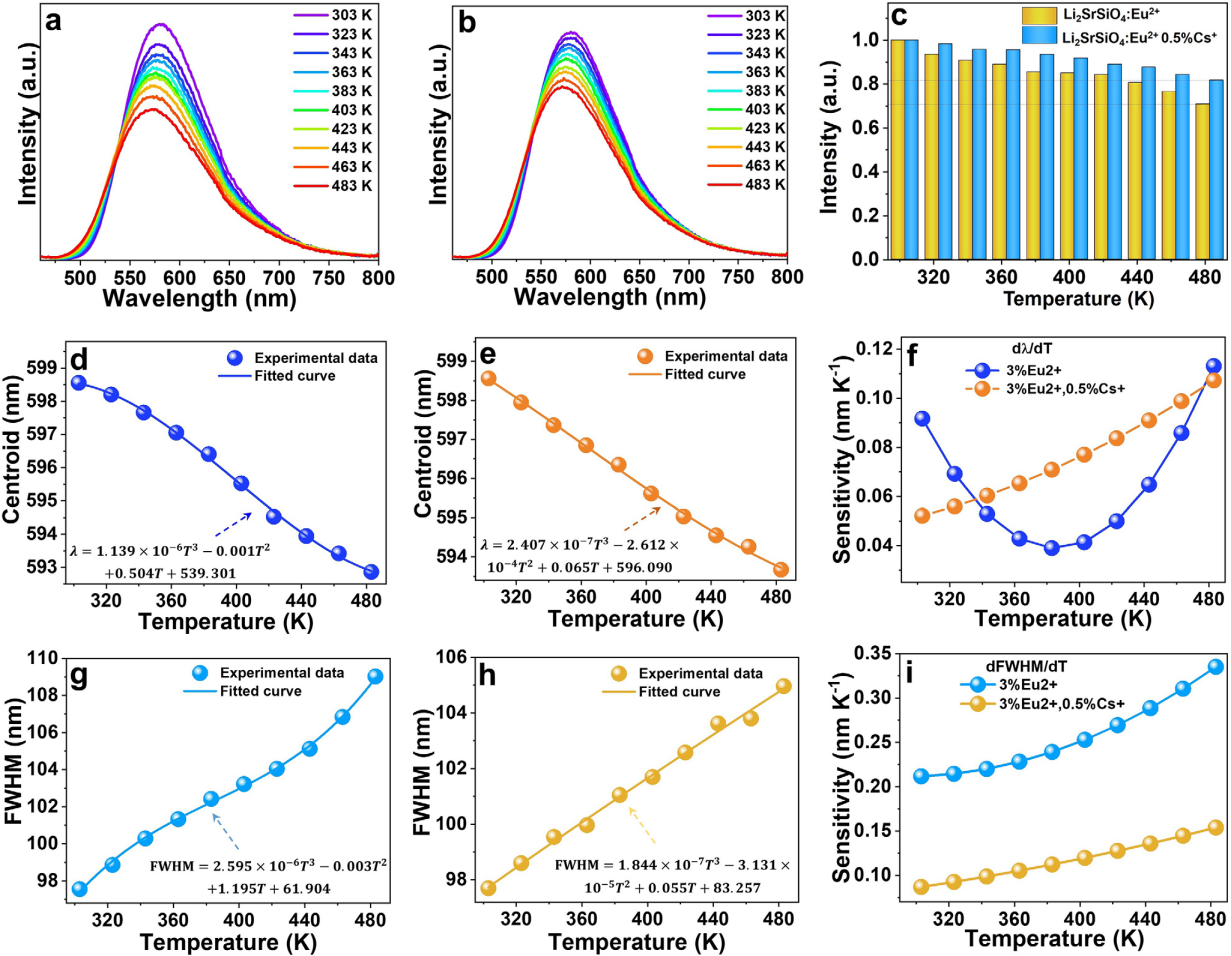
(a,b) Temperature‐dependent emission spectra of the Li_2_SrSiO_4_:3%Eu^2+^ (a) and Li_2_SrSiO_4_:3%Eu^2+^,0.5%Cs^+^ (b) phosphors; (c) Normalized luminescence intensities of the obtained phosphors as a function of temperature. (d‐f) Temperature‐dependent emission band centroid of the Eu^2+^‐doped (d), and Eu^2+^,Cs^+^‐codoped Li_2_SrSiO_4_ (e), and absolute sensitivities of phosphors (f). (g–i) Temperature‐dependent FWHM of the Eu^2+^‐doped (g), and Eu^2+^,Cs^+^‐codoped Li_2_SrSiO_4_ (h), and sensitivities of phosphors (i).

To ensure stable performance of the fabricated white‐LED under operating conditions, and to extend the applications of the studied sample as a temperature sensor, the thermal stability and temperature‐dependent emission spectra of the phosphors were studied at elevated temperature conditions. The emission spectra of the Li_2_SrSiO_4_:3%Eu^2+^ and Li_2_SrSiO_4_:3%Eu^2+^,0.5%Cs^+^ phosphors recorded in the temperature range of 303–483 K are presented in Figure 3a,b, respectively. As displayed, with increasing temperature, the luminescence intensities decrease for both materials due to the thermal quenching effects. Specifically, when the temperature is 423 K, the luminescence intensity of the Li_2_SrSiO_4_:3%Eu^2+^ phosphor keeps its starting value of 84.2% at 303 K, while that of the Li_2_SrSiO_4_:3%Eu^2+^,0.5%Cs^+^ phosphor preserves even higher intensity of 88.9% (see Figure 3c), confirming the beneficial impact of Cs^+^ doping and the remarkable thermal stability of these materials, enabling their applications as white‐LED and temperature sensors. Moreover, through analyzing the relation between the luminescence intensity and temperature, the activation energy of the studied samples is calculated, as Equation (5) defined below: [70]

(5) $$I=\frac{I_{0}}{1+Aexp\left(-\Delta E/kT\right)}\;$$

where *I_0_* and *I* are the luminescence intensities of the phosphors at initial and recorded temperatures, respectively, *A* is the constant, *ΔE* is the activation energy, and *k* is the Boltzmann constant. The plots of ln(*I_0_*/*I*‐1) vs. 1/*kT* for the resultant compounds are shown in Figure S3. As disclosed, these experimental data can be linearly fitted, and the *ΔE* values of the Li_2_SrSiO_4_:3%Eu^2+^ and Li_2_SrSiO_4_:3%Eu^2+^,0.5%Cs^+^ phosphors are calculated as 0.133 and 0.199 eV, respectively.

Importantly, apart from the quenched luminescence, it is also found that emission band position and bandwidth (*i.e*. full width at half maximum—FWHM) are temperature dependent. As demonstrated in Figure 3d, the emission band centroid of the Li_2_SrSiO_4_:3%Eu^2+^ phosphors blueshifts from 598.5 to 592.8 nm as the temperature rises from 303 to 483 K. The relation between temperature and emission band centroid can be expressed as: *λ* = 1.139 × 10^−6^ *T* ^3^ – 0.001*T* ^2^ + 0.504*T* + 539.301. On the other hand, emission band centroid for the Li_2_SrSiO_4_:3%Eu^2+^,0.5Cs^+^ phosphor also blueshifts with temperature, changing from 598.5 to 593.7 nm within the applied temperature range of 303–483 K, and its temperature evolution can be described as: *λ* = 2.407 × 10^−7^ *T* ^3^ – 2.612 × 10^−4^ *T* ^2^ + 0.065*T* + 596.090 (Figure 3e). Since the centroids of the emission bands exhibit strong temperature dependence, optical temperature sensing can be achieved by employing this parameter as a temperature indicator. To describe the thermometric properties of the investigated materials, the absolute sensitivity (i.e., *S_a_*) was calculated according to the following Equation (6):

(6) $$S_{a}=\frac{dMP}{dT}\;$$

where *MP* is the measured parameter (i.e. FWHM or emission band centroid). Accordingly, the temperature‐dependent *S_a_* values for the Li_2_SrSiO_4_:3%Eu^2+^ and Li_2_SrSiO_4_:3%Eu^2+^,0.5%Cs^+^ phosphors, in which the emission band centroids are used as thermometric parameters, are obtained and shown in Figure 3f. Apparently, the *S_a_* values are dependent on temperature, and their maximal values were determined as 0.113 and 0.107 nm K^−1^ for each emission band centroid, respectively. The FWHM (Figure 3g) for the Li_2_SrSiO_4_:3%Eu^2+^ shows an opposite tendency than band centroid, i.e., it increases from 97.5 to 109.1 nm with temperature elevation, and the relation between temperature and FWHM can be described as FWHM = 2.595 × 10^−6^ *T* ^3^ – 0.003*T* ^2^ + 1.195*T* + 61.904. In contrast, the FWHM parameter, for Li_2_SrSiO_4_:3%Eu^2+^,0.5%Cs^+^ phosphor increases from 97.7 to 104.9 nm as temperature rises from 303 to 483 K (see Figure 3h), which can be correlated with temperature as FWHM = 1.844 × 10^−7^ *T* ^3^ – 3.131 × 10^−5^ *T* ^2^ + 0.055*T* + 83.257. In addition, according to the Equation 6, the *S_a_* values for the Eu^2+^‐doped and Eu^2+^,Cs^+^‐co‐doped phosphors are estimated as 0.335 and 0.154 nm K^−1^, respectively, when the FWHM and emission band centroid are employed as the thermometric parameters (Figure 3i). Compared with those of the Li_2_SrSiO_4_:3%Eu^2+^ phosphors, the Li_2_SrSiO_4_:3%Eu^2+^,0.5Cs^+^ phosphors exhibit smaller *S_a_* values, which may result from the thermal stabilization of luminescence intensity and lower probability of non‐radiative relaxation. These results suggest that the Li_2_SrSiO_4_:3%Eu^2+^ phosphor with higher sensitivity is more suitable for optical thermometry compared with the compounds co‐doped with Eu^2+^ and Cs^+^ ions.

### 2.3 Pressure‐Dependent Properties

**FIGURE 4:**
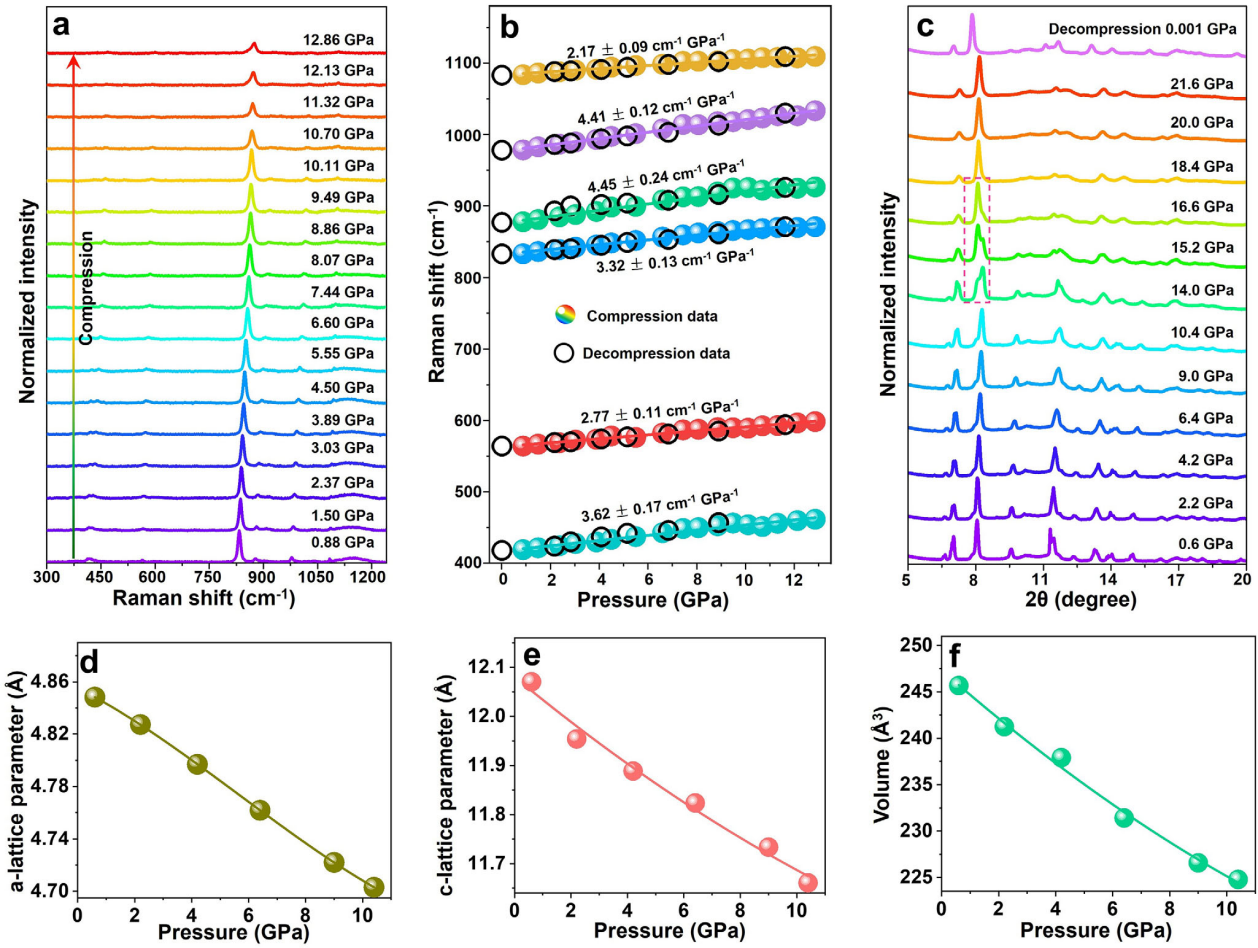
(a,b) Pressure‐dependent Raman spectra (a) and determined Raman peak positions (b) of the Li_2_SrSiO_4_ material. (c) XRD patterns of the Li_2_SrSiO_4_:3%Eu^2+^,0.5%Cs^+^ material collected as a function of pressure. (d–f) Pressure evolution of the unit‐cell dimmensions (d,e) and unit‐cell volume (f) of the Li_2_SrSiO_4_:3%Eu^2+^,0.5%Cs^+^ material.

The structural stability of the optimized material under high‐pressure conditions was characterized by the in situ pressure‐dependent Raman spectroscopy and high‐pressure XRD experiments. Figure 4a shows the Raman spectra of the Li_2_SrSiO_4_ matrix in the pressure range between 0.88 and 12.86 GPa. The measured Raman spectra contain six Raman peaks located at 419, 564, 833, 878, 978 and 1084 cm^−1^. According to previous reports [27, 30], the vibration modes at the frequencies of 419 and 564 cm^−1^ are attributed to the rotation of the LiO_4_ tetrahedron, while the other vibration modes with the higher frequencies are assigned to the bending and stretching modes of the SiO_4_ tetrahedron. It can be seen from Figure 4a that all Raman modes gradually shift to higher wavenumbers, which is caused by the shortening of bond lengths (decrease of interatomic distance) throughout the whole compression cycle. When the studied sample undergoes the decompression process (see Figure S4), the Raman bands return to their initial positions, implying that the hexagonal phase of Li_2_SrSiO_4_ material has good structural stability under extreme stress conditions, and it is preserved up to 12.86 GPa . Furthermore, the Raman modes initially present at 419, 564, 833, 878, 978, and 1084 cm^−1^ are shifted with 3.62 ± 0.17, 2.77 ± 0.11, 3.32 ± 0.13, 4.45 ± 0.24, 4.41 ± 0.12, and 2.17 ± 0.09 cm^−1^ GPa^−1^ rates, respectively (Figure 4b). Note that, aside from the shift of the Raman modes, it is also observed that the Raman peaks are widened, and the signal‐to‐noise ratio in the Raman spectra decreases, which is triggered by the increased amount of crystal defects and strains in the compressed material.

Further investigation of the in situ pressure‐dependent XRD patterns was carried out to show the impact of high pressure on the phase stability of the investigated Li_2_SrSiO_4_:3%Eu^2+^,0.5%Cs^+^ phosphor; the corresponding results are shown in Figure 4c. Note that the positions of the diffraction reflexes presented in Figure 4c are different from those presented in Figure 1a due to the different wavelength of the radiation source used for the high‐pressure XRD measurements, i.e., Ag *Kα* radiation (*λ* = 0.4834 Å). As disclosed, when pressure is lower than 14 GPa, the diffractograms of the studied sample are similiar, except for the shift of the diffraction peaks to the higher 2θ angles due to decreasing interatomic distances, implying no phase transition in the investigated material below that pressure. However, a noticable change in high‐pressure diffractograms above 14.0 GPa indicate a pressure‐induced phase transition. Specifically, the 14.0–15.2 GPa range was identyfied as a coexistence range of ambient‐pressure hexagonal  phase and the newly emerged high‐pressure phase β. Above 16.6 GPa, the studied material exists only as a triclinic phase β. Moreover, with further increase of pressure (i.e. 16.6 ≤ *p* ≤ 21.6 GPa), the diffraction peaks are furher shifted to a higher 2θ angles, indicating pressure‐induced lattice shrinkage of the newly formed material.

Notably, when the pressure is released, the material does not return to its initial hexagonal structure, but instead it remains in the high‐pressure triclinic phase (see Figure 4c), suggesting that the new material structure formed under extreme conditions remains stable at ambient conditions. Benefiting from these results, the phosphor obtained at high‐pressure may exhibit unique pressure‐tailored luminescence properties. Since no phase transition occurs in the studied sample at relatively low pressure, the compression of phase α leads to monotonic changes of unit‐cell dimmensions, as shown in Figure 4d–f. The lattice parameters (i.e., *a* = *b*, *c*, and *V*) decrease monotonically, due to the lattice shrinkage of the material unit cell during the compression process. Furthermore, the bulk compressibility of the phase α can be quantitatively described by the second‐order Birch–Murnaghan equation of state Equation (7), as defined below: [71]

(7) $$P\;\left(V\right)=\frac{3B_{0}}{2}\;\left[{\left(\frac{V_{0}}{V}\right)}^{7/3}-{\left(\frac{V_{0}}{V}\right)}^{5/3}\right]$$

Accordingly, the *B_0_* value is decided to be 86.94 GPa.

**FIGURE 5:**
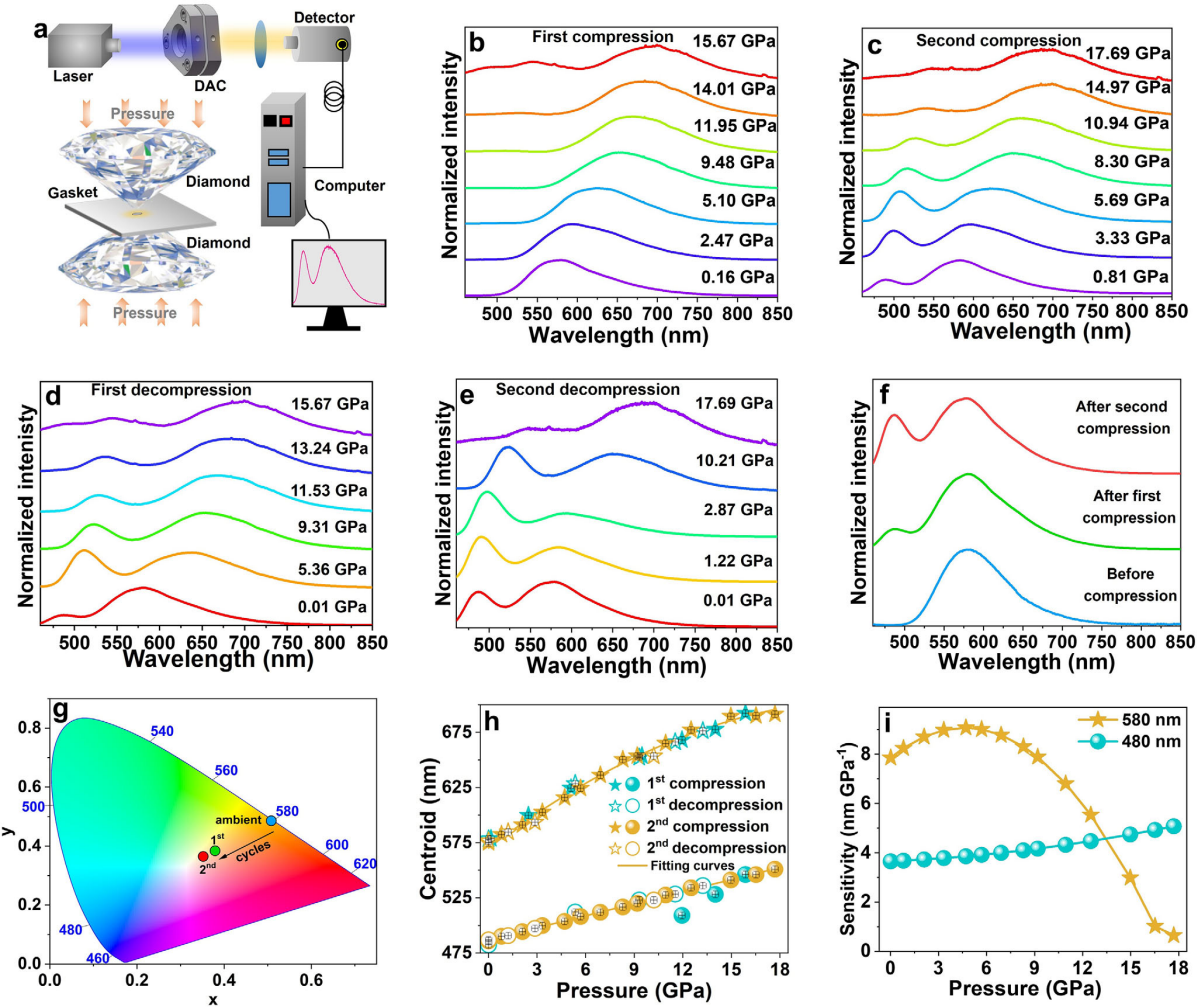
(a) The experimental setup scheme used for high‐pressure measurements. (b,c) Normalized emission spectra of the Li_2_SrSiO_4_:3%Eu^2+^,0.5%Cs^+^ phosphor during the first (b) and second (c) compression cycle, excited at 450 nm. (d,e) Emission spectra of the Li_2_SrSiO_4_:3%Eu^2+^,0.5%Cs^+^ phosphor during the first (d) and second (e) decompression cycle. (f) Comparison of the emission spectra for the investigated material measured at ambient conditions, before compression, after the first and second compression–decompression cycles. (g) CIE diagram with comparison of visible emission color before and after both compression–decompression cycles. (h) Pressure‐dependent emission band centroids of the Li_2_SrSiO_4_:3%Eu^2+^,0.5%Cs^+^ phosphor during both compression–decompression processes. (i) Calculated pressure sensitivity for both emission bands plotted as a function of pressure.

In an attempt to reveal the evolution of the luminescence properties of the studied samples, their in situ pressure‐dependent emission spectra were measured in a DAC, as presented in Figure 5a. Here, a 450 nm laser diode was used as the excitation source. The pressure‐dependent emission spectra of the Li_2_SrSiO_4_:3%Eu^2+^,0.5%Cs^+^ phosphor during the first compression process are presented in Figure 5b. As presented, when the pressure is lower than 14.01 GPa, the studied material only shows a single broad band, which redshifts as pressure increases. However, when the pressure rises to 15.67 GPa, a new high‐energy emission band, located at around 550 nm, emerges alongside the initial band. Importantly, during the second compression cycle (up to 17.69 GPa), it was found that the emission spectrum initially (at ambient conditions) consists of two broad bands, centered at around 480 and 580 nm, both exhibiting a significant redshift with pressure, as presented in Figure 5c. All emission spectra of the second compression cycle are presented in Figure S5. It is clear that the luminescence properties of the investigated material can be tailored via high‐pressure engineering. Moreover, when the synthesized material undergoes the first decompression cycle (Figure 5d), its luminescence features do not recover to the initial state, i.e., two broad emission bands are observed during the decompression process, and changes are irreversible. In contrast, during the second decompression process, the emission spectra can be recovered, that is, the position of the bands is the same as before the second compression process (Figure 5e), however the intensity of the band at 480 nm relatively increases. These results suggest that the pressure‐induced changes in the material PL are permanent, and they can be maintained even after the pressure is released. This effect is caused by a change in the local symmetry around Eu^2+^ ions and by the presence of Eu^2+^ occupying two non‐identical sites in the newly formed crystal structure. This also confirms the occurrence of a phase transition under pressure, which agrees well with the in situ high‐pressure XRD data. For clarity, the emission spectra before and after the first and second compression‐decompression cycles are compared in Figure 5f, where the pressure‐induced appearance and intensity enhancement of the new emission band at 480 nm are clearly observed. The newly formed emission band centered at 480 nm exhibits a substantially narrower profile compared to the original band at 580 nm, with FWHM values of approximately 35 and 102 nm, respectively. Based on the recorded spectra, the observed color of emission was compared in the CIE diagram in Figure 5g. The chromaticity coordinates shift toward the center of the CIE diagram during the pressure‐induced phase transition of the material, as a result of the local symmetry changes around the Eu^2+^ ions.

Based on the above experimental results, the origin of the observed structural and spectroscopic changes can be discussed. The initial structural changes observed in Raman, photoluminescence, and XRD up to 12.86 GPa i.e., peak shifts and broadening, are attributed to pressure‐induced lattice strain formation, as no new modes or diffraction peaks are detected, and the changes are reversible upon decompression. At pressure above 14 GPa, a structural transformation from the hexagonal phase α to triclinic phase β occurs, indicating that the stability limit of the initial hexagonal phase has been reached. This transition occurs via strain‐induced lattice reorganization and is associated with defect formation, confirmed by the appearance of a new emission band at 480 nm. Furthermore, the coexistence of both phases in the pressure range of 14–16.6 GPa suggests a gradual growth of a new, triclinic phase, promoting defect formation at phase boundaries.

It is well known that the luminescence properties of Eu^2+^ are highly dependent on crystal field splitting, Stokes shift, and nephelauxetic effect, where the crystal field splitting takes the dominant role [25, 72, 73]. In general, via the utilization of the following function, the crystal field splitting (*D_q_*) can be estimated with Equation (8): [74]

(8) $$D_{q}=\frac{1}{6}\;Ze^{2}\frac{r^{4}}{R^{5}}$$

where *Z* stands for the charge of the anion, *e* refers to the elementary charge, *r* is the radius of the wave function, and *R* is the bond length between the central atom and ligand. As is known, the values of *Z*, *e*, and *r* are fixed, and thus, the *D_q_* value is inversely proportion to *R* ^5^. Consequently, the lattice contraction will result in the shortened bond lengths, as well as the increased *D_q_* value, contributing to the redshift of the emission band. Additionally, the average distance between O^2−^ and Eu^2+^ ions will also decrease at high‐pressure, which enhances the nephelauxetic effect and leads to a stronger covalent character of the bonds. As a result, the energy of the lowest excited 5*d* level of Eu^2+^ ions will be decreased, causing the spectral redshift in the investigated phosphor. Furthermore, Stokes shift (i.e., *E*_Stokes_), which is related to the energy difference between the maxima of the emission and excitation bands, can be evaluated by means of the following function from Equation (9): [75]

(9) $$E_{\mathrm{Stokes}}=\left(2S-1\right)\;hv$$

where *hv* and *S* refer to the phonon energy and Huang–Rhys coupling, respectively. When the studied sample is subjected to high‐pressure compression, the atoms will depart from their equilibrium states, increasing the crystal and potential energies, and leading to higher phonon energy in the compressed material. The increased energy of the system enhances electron–phonon interactions, leading to faster non‐radiative relaxation and energy loss, consequently, a larger Stokes shift (E_s__t__o__k__e__s_). A distinct redshift is observed in the studied material owing to the synergistic effect of these factors.

Since the emission band position is highly dependent on pressure, the studied phosphor can be used in optical manometry. Herein, the emission band centroids were adopted as the manometric parameters for the pressure detection. Based on the recorded emission spectra during the first and second compression–decompression processes, the pressure‐dependent emission band centroid was calculated and shown in Figure 5h. As the pressure rises from 0.16 to 15.67 GPa, the emission band centroid shifts from 578.1 to 692.3 nm. Note that the data calculated for the band at 475 nm from the first compression process deviates from the further results, which is due to the pressure‐induced phase transition effect and formation of the blue emission band under the first compression process. This characteristic behavior makes the data obtained from the first compression–decompression processes are unsuitable for optical manometry. Moreover, based on the emission spectra of the Li_2_SrSiO_4_:3%Eu^2+^,0.5%Cs^+^ material measured during the second compression–decompression processes, the pressure‐dependent emission band centroids were calculated and presented in Figure 5h. In this case, the decompression data points match well with the compression data, of which band positions of the blue (λ ≈ 480 nm) and yellow (λ ≈ 580 nm) both exhibit redshift tendency with pressure, i.e. the blue emission band shifts from 486.1 to 550.9 nm, while the yellow band shifts from 582.4 to 692.3 nm, as the pressure rises from 0.81 to 17.69 GPa. By analyzing the experimental data, the relation between the blue emission band centroid and pressure was established with an empirical function: *λ* = ‐9.53 × 10^−4^ *p* ^3^ + 0.015*p* ^2^ + 3.65*p* + 486.6, while for the yellow emission band it could be expressed as *λ* = ‐0.018*p* ^3^ + 0.264*p* ^2^ + 7.85*p* + 574.9.

To describe the manometric properties of the developed sensor materials, the pressure sensitivity, i.e. d*λ*/d*p*, was determined as shown in Figure 5i. When the band centroid of the blue emission is adopted as the manometric parameter, the pressure sensitivity shows an upward trend with pressure, reaching its maximum value of 5.07 nm GPa^−1^ at 17.69 GPa. Whereas using the yellow emission band shift, the maximum pressure sensitivity reaches 9.08 nm GPa^−1^ at 4.71 GPa. The calculated pressure sensitivities are larger than those of most previously developed luminescent manometers, as listed in Table 1, and they are ≈14 and 25 times greater, respectively, than the sensitivity of the commonly used ruby sensor (whose d*λ*/d*p* = 0.365 nm GPa^−1^). According to the performed PL studies, the developed sensors can operate well in a relatively wide pressure range, i.e., below 18 GPa (or even higher, as suggested by the XRD data), which is superior to the majority of the previous reports [58, 76, 77, 78]. It is worth noting that the developed material, subjected to high‐pressure postsyntetic treatment, exhibits markedly improved properties, i.e., an additional, narrow emission band of the new high‐pressure phase, which linearly shifts with pressure, significantly enhancing its manometric performance for precise optical sensing application.

**TABLE 1:** Comparison of the optical pressure sensing performance of the optical manometers based on the emission band shift, operating in a visible range.

Phosphor	Transition	*λ*_em_ (nm)	Operating range (GPa)	*S_a(MAX)_* (nm GPa^−1^)	Ref.
Li_2_SrSiO_4_:3%Eu^2+^,0.5%Cs^+^	*4f^6^5d^1^→4f^7^*	575 475	17.69	9.08 5.07	This work
Cs_2_Ag_0.6_Na_0_._4_InCl_6_:Bi^3+^	exciton‐type	600	3.87	−112	[58]
SrB_4_O_7_:Tm^2+^	*4f^12^5d→4f^13^*	600	12.74	0.43	[78]
Eu‐MOF	*^5^D_0_→^7^F_4_*	694	10.47	0.36	[79]
ZnS/CaZnOS:Mn^2+^	*^4^T_1_→^6^A_1_*	618	19.20	6.20	[50]
Sr_4_Al_14_O_25_:Mn^4+^	*^2^E→^4^A_2_*	655	7.59	1.2	[80]
Sr_8_Si_4_O_12_Cl_8_:Eu^2+^	*4f^6^5d^1^→4f^7^*	497	7.35	9.69	[26]
AlN	defect‐type	681	18	18.5	[81]
Zn_2_GeO_4_:Mn^2+^	*^4^T_1_(^4^G)→^6^A_1_*	535	6.76	21.3	[82]
Ca_9_NaZn(PO_4_)_7_:Eu^2+^	*4f^6^5d^1^→4f^7^*	570	16.48	5.21	[83]
Li_4_SrCa(SiO_4_)_2_:Eu^2+^	*4f^6^5d^1^→4f^7^*	584	10	5.19	[76]
SrB_4_O_7_:Eu^2+^,Sm^2+^	*^6^P_7/2_→^8^ S_7/2_*	362	34.57	0.17	[24]
Lu_2_Mg_2_Al_2_Si_2_O_12_:Eu^2+^,Mn^2+^	*4f^6^5d^1^→4f^7^*	472	10	1.77	[84]
BaCN_2_:Eu^2+^	*4f^6^5d^1^→4f^7^*	660	5.34	19.0	[37]
BaLi_2_Al_2_Si_2_N_6_:Eu^2+^	*4f^6^5d^1^→4f^7^*	532	20.11	1.58	[75]
Sr_2_MgSi_2_O_7_:Eu^2+^,Dy^3+^	4f^6^5d^1^ → 4f^7^	460	≈10	8.11	[77]

### 2.4 Formation of New Material in a Large‐Format, High‐Pressure Multi‐Anvil Press

**FIGURE 6:**
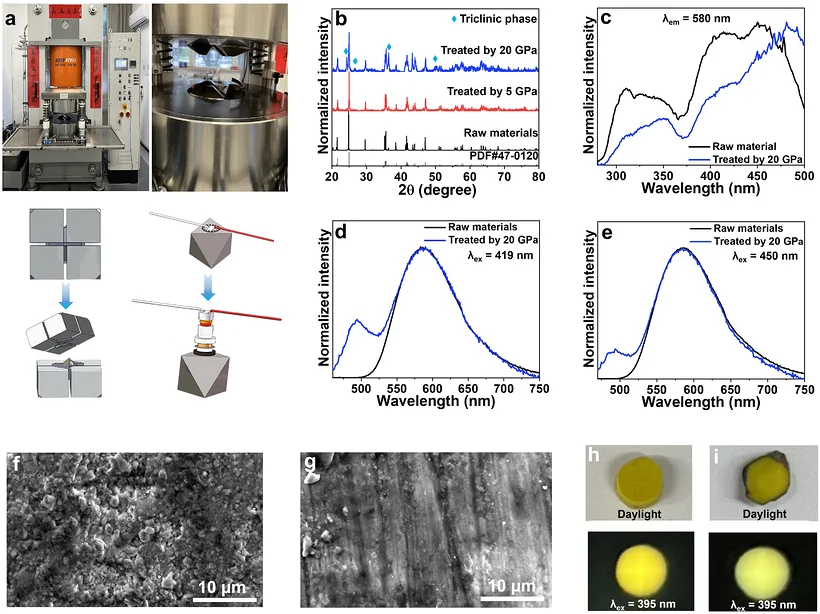
(a) Schematic representation of the large‐format high‐pressure press (bottom), together with the corresponding photographs of the multi‐anvil cell device used (top). (b) XRD patterns of the materials, untreated and post‐treated under different high‐pressure conditions in a large‐format press. (c) Normalized excitation spectra of the prepared phosphors (λ_em_ = 580 nm). (d,e) Normalized emission spectra of the as‐synthesized and pressure‐treated phosphors, excited at different wavelengths λ_ex_ = 419 nm (d) and λ_ex_ = 450 nm (e). (f,g) SEM images of the compressed Li_2_SrSiO_4_:3%Eu^2+^,0.5%Cs^+^ phosphor pellets treated by different pressures of 5 GPa (f) and 20 GPa (g). (h,i) Photographs of the studied samples treated at 5 GPa (h) and 20 GPa (i), taken under daylight and 395 nm UV light excitation.

The optimized Li_2_SrSiO_4_:3%Eu^2+^,0.5%Cs^+^ phosphor was subjected to two separate compression–decompression cycles in a large‐format, high‐pressure multi‐anvil press, which allows for compressing millimetric volume (∼22.6 mm^3^) of the material during a single compression cycle. The corresponding device (top) and high‐pressure press operation process (bottom) are shown in Figure 6a. A high‐pressure multi‐anvil press operates by gradually compressing a sample placed in a central capsule using a set of outer anvils that evenly transmit force, enabling the generation of very high static pressures under controlled loading conditions [51, 65, 66, 85, 86]. The first batch of the sample was compressed up to 5 GPa for 12 h, and then the pressure was released, while another batch was compressed up to 20 GPa for 12 h and subsequently decompressed. After pressure release, the materials were collected for further investigation. The XRD patterns of the initial Li_2_SrSiO_4_:3%Eu^2+^,0.5%Cs^+^ phosphor and two compressed compounds were measured and presented in Figure 6b. As displayed, when the studied phosphor was compressed to 5 GPa, its diffraction pattern (upon pressure release) is the same as the initial Li_2_SrSiO_4_:3%Eu^2+^,0.5%Cs^+^ material, suggesting that the relatively low pressure cannot induce the phase transition, which agrees with previous studies from the DAC (see Figure 4c). Nevertheless, when the pressure was increased to 20 GPa, a new crystal structure corresponding to the high‐pressure triclinic β‐phase appeared, coexisting with the hexagonal phase of Li_2_SrSiO_4_ in the recovered material. These results suggest that the high‐pressure is an efficient route to induce the phase transition and permanently modify the studied phosphor material. To check the PL properties of the newly formed material, its excitation and emission spectra were measured. As presented in Figure 6c, there is an irreversible change of the excitation band shape for the material compressed to 20 GPa, compared to the uncompressed one. Furthermore, the emission spectra also show the irreversible transformation of the material, as a formation of a new emission band at around 480 nm (Figure 6d,e), which agrees with the PL data from the DAC (see Figure 5b–f). The relative intensities of all emission spectra were compared in Figure S6. The observed alterations in the PLE and PL spectra are mainly due to the formation of the new phase with different site symmetry of Eu^2+^ ions, leading to the coexistence of two different sites for the emitting ions. Additionally, the occurrence of plastic (inelastic) deformations in the initial material structure may contribute to the observed changes, as well. From the recorded SEM images (Figure 6f,g), it can be seen that the compressed material grains become more densely packed, with a smoother surface, implying the effect of pressure on their morphology, as well. Additionally, the sample treated by lower pressure can still emit bright yellowish light (Figure 6h,i). Notably, the material subjected to pressure above 20 GPa exhibits more white PL color (see Figure 5g) due to the appearance of a new high‐energy band located in the blue‐cyan range. Those results confirm the permanent change of the Li_2_SrSiO_4_ crystal structure under high‐pressure conditions and the formation of a novel high‐pressure phase with unique PL properties.

## 3 Conclusions

Here we show, for the first time, the use of a large‐format high‐pressure press for post‐treatment of the synthesized phosphors, leading to the formation of new materials with unique optical properties, for the development of new white‐LED devices and optical manometers with improved performance. A series of Cs^+^,Eu^2+^ co‐doped Li_2_SrSiO_4_ phosphors was successfully synthesized using the high‐temperature solid‐state synthetic protocol. When excited at 450 nm, the resulting phosphors exhibit the characteristic emission of Eu^2+^ ions, which can be enhanced by the introduction of Cs^+^ ions, leading to the improved PL intensity, thermal stability, and higher quantum efficiency. The designed phosphors, i.e., Li_2_SrSiO_4_:3%Eu^2+^,0.5%Cs^+^, were further used as yellow‐emitting energy converters for the development of a white‐LED and optical thermometry.

Subsequently, the optimized phosphor was subjected to high‐pressure conditions (up to ≈20 GPa) in a DAC, and by means of in situ Raman scattering, synchrotron XRD, and PL spectroscopy, its structural stability and optical properties under extreme conditions were explored. The performed studies enabled the development of a novel, efficient optical manometer operating in a band‐shift mode. Noteworthy, above 14.0 GPa a new high‐pressure phase has been formed, which exhibits unique optoelectronic properties, such as pressure‐induced emission (appearance of a new high‐energy component), improved white‐LED characteristics, and enhanced pressure sensing performance. Most importantly, we showed that the utilization of a macro‐scale high‐pressure engineering, i.e. the use of a large‐format press vs. a DAC device, is an efficient route to form new materials stable at room conditions (via a pressure‐induced, permanent phase transition), which exhibit unique and modified luminescence features. This strategy also enables the facile fabrication and collection of a large amount of pressure‐treated material, in contrast to the DAC device, allowing for its real‐world applications in white‐LED and optical manometry.

## 4 Experimental Section

### 4.1 Synthesis

SrCO_3_ (99.5%, Sigma–Aldrich), Li_2_CO_3_ (97%, Sigma–Aldrich), SiO_2_ (99.99%, Sigma–Aldrich), Eu_2_O_3_ (99.9%, Stanford Materials), and Cs_2_CO_3_ (99.9%, Sigma–Aldrich) were used as precursors for materials synthesis. The solid‐state synthesis was used to synthesize the Li_2‐2x_Sr_0.97_SiO_4_:0.03Eu^2+^,2*x*Cs^+^ (*x* = 0.003, 0.005, 0.007, 0.01, 0.02, and 0.03) phosphors. The stoichiometric amounts of the SrCO_3_, Li_2_CO_3_, SiO_2_, Eu_2_O_3_, and Cs_2_CO_3_ powder were mixed thoroughly in a mortar for 10 min. Grounded precursors were transferred to a crucible and calcined at 1073 K for 6 h. The final materials were grounded to a powder for further investigations.

### 4.2 Characterization

Powder X‐ray diffraction patterns (XRD) at ambient conditions were recorded using a Bruker AXS D8 Advance diffractometer in Bragg–Brentano geometry, with Cu Kα1 radiation (*λ* = 1.5406 Å) and 0.05° step scan mode. The reference pattern for comparison was taken from ICSD (Inorganic Crystal Structure Database). Scanning electron microscopy (SEM) images were taken with a scanning electron microscope FEI Quanta 250 FEG equipped with an EDAX detector. The luminescence properties of the materials, i.e., emission spectra at room‐ and high‐temperature, were investigated using an Andor Shamrock 500i spectrometer with a silicon CCD iDus camera as a detector. Materials were excited using a 450 nm continuous wave (CW) semiconductor blue laser with a power of 50 mW. The measurements were corrected for the apparatus response. The varied temperature conditions were generated with the Linkam THMS 600 heating‐cooling stage. XRD patterns at high‐pressure conditions were recorded in DAC for 12 h with silicon oil using synchrotron radiation of *λ* = 0.4834 Å. High‐pressure luminescence measurements were done in a Merill‐Basset type DAC equipped with IIas type low fluorescent diamonds. Methanol‐ethanol‐water (16:3:1) was used as a pressure‐transmitting medium. The Ruby (Al_2_O_3_:Cr^3+^) microspheres with ∼20 µm diameter were used as a pressure indicator using the R_1_ line of Cr^3+^ emission. Scattering Raman spectra were recorded in the varied pressure conditions in a backscattering geometry using a Renishaw InVia confocal micro‐Raman system with a 100 mW 532 nm diode laser as excitation source and an objective Olympus x20 SLMPlan N long working distance to focus the laser beam on the material surface. Materials were compressed in a high‐pressure multi‐anvil large‐formatting press Mavo LPR1000‐400/50 to 20 GPa to prepare compressed pellets in the cylindrical sample holder with 1.2 mm in diameter and 1.5 mm in height.

## Conflicts of Interest

The authors declare no conflicts of interest.

## Supporting information




**Supporting File**: advs75887‐sup‐0001‐SuppMat.docx.

## Data Availability

The data that support the findings of this study are available from the corresponding author upon reasonable request.
