# Supplementary material for: Pressure‐Driven Phase Transition Unlocking Unique Eu2+ Luminescence in Li2SrSiO4 for Optical Sensing and White‐LEDs
Source: Adv Sci (Weinh). 2026 Jun 2:e75887. Online ahead of print. doi: 10.1002/advs.75887 (PMC13336095; doi:10.1002/advs.75887)
Supplement: Supplementary file 1 — Supporting File: advs75887‐sup‐0001‐SuppMat.docx. [file ADVS-9999-e75887-s001.docx]

**Pressure-Driven Phase Transition Unlocking Unique Eu^2+^ Luminescence in Li_2_SrSiO_4_ for Optical Sensing and White-LEDs**

Przemysław Woźny^*^, Peng Du^*^, Teng Zheng, Junpeng Xue, Liang Peng, Shuailing Ma^*^, Szymon Sobczak, Victor Lavín, Tian Cui, Marcin Runowski^*^

P. Woźny, T. Zheng, L. Peng

School of Information and Electrical Engineering, Hangzhou City University, Hangzhou, Zhejiang, China

P. Woźny, S. Sobczak, M. Runowski

Adam Mickiewicz University, Faculty of Chemistry, Uniwersytetu Poznańskiego 8, 61-614 Poznań, Poland

*E-mail: przemyslaw.wozny@amu.edu.pl (P. Woźny), runowski@amu.edu.pl (M. Runowski),

P. Du, S. Ma, T. Cui

School of Physical Science and Technology, Ningbo University, 315211 Ningbo, Zhejiang, China

*E-mail: dupeng@nbu.edu.cn (P. Du); mashuailing@nbu.edu.cn (S. Ma)

V. Lavín

Departamento de Física, MALTA-Consolider Team & IUdEA, Universidad de La Laguna, Apartado de Correos 456, E-38200 San Cristóbal de La Laguna, Santa Cruz de Tenerife, Spain

J. Xue

School of Science, Jiangsu University of Science and Technology, Zhenjiang, 212100, China

**Table S1** lattice parameter of the Li_2_SrSiO_4_:3%Eu^2+^ and Li_2_SrSiO_4_:3%Eu^2+^,0.5%Cs^+^ phosphors

| Parameter | Compounds | |
| --- | --- | --- |
|  | Li_2_SrSiO_4_:3%Eu^2+^ | Li_2_SrSiO_4_:3%Eu^2+^,0.5%Cs^+^ |
| *a* = *b* | 5.02613 Å | 5.02616 Å |
| *c* | 12.46206 Å | 12.46306 Å |
| *V* | 272.661 Å^3^ | 272.664 Å^3^ |
| *R_wp_* | 13.88% | 13.57% |
| *R_p_* | 9.37% | 9.78% |
| χ^2^ | 1.50 | 1.32 |

**Table S2** EL properties of the packaged white-LED as a function of driving current.

| Current | CCT | CRI | Color coordinate | Efficiency |
| --- | --- | --- | --- | --- |
| 60 mA | 5674 K | 75 | (0.330,0.253) | 105 lm/W |
| 80 mA | 5832 K | 75 | (0.329,0.253) | 102 lm/W |
| 100 mA | 5920 K | 75 | (0.328,0.253) | 100 lm/W |
| 120 mA | 6022 K | 76 | (0.327,0.253) | 98 lm/W |
| 140 mA | 6028 K | 76 | (0.327,0.254) | 94 lm/W |
| 160 mA | 6094 K | 76 | (0.326,0.254) | 92 lm/W |
| 180 mA | 6196 K | 76 | (0.325,0.254) | 93 lm/W |
| 200 mA | 6244 K | 76 | (0.325,0.254) | 90 lm/W |


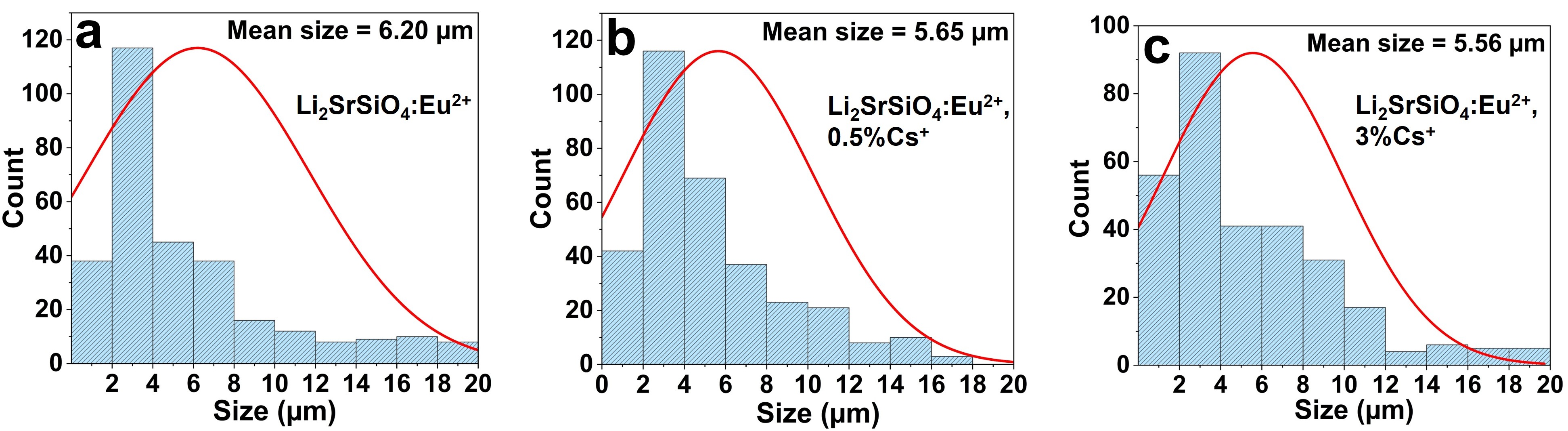


**Figure S1** Histograms showing the particle size distribution for the corresponding SEM images of the Li_2_SrSiO_4_:3%Eu^2+^,xCs^+^ phosphors with the x value of (a) 0.0%, (b) 0.5% and (c) 3.0%.


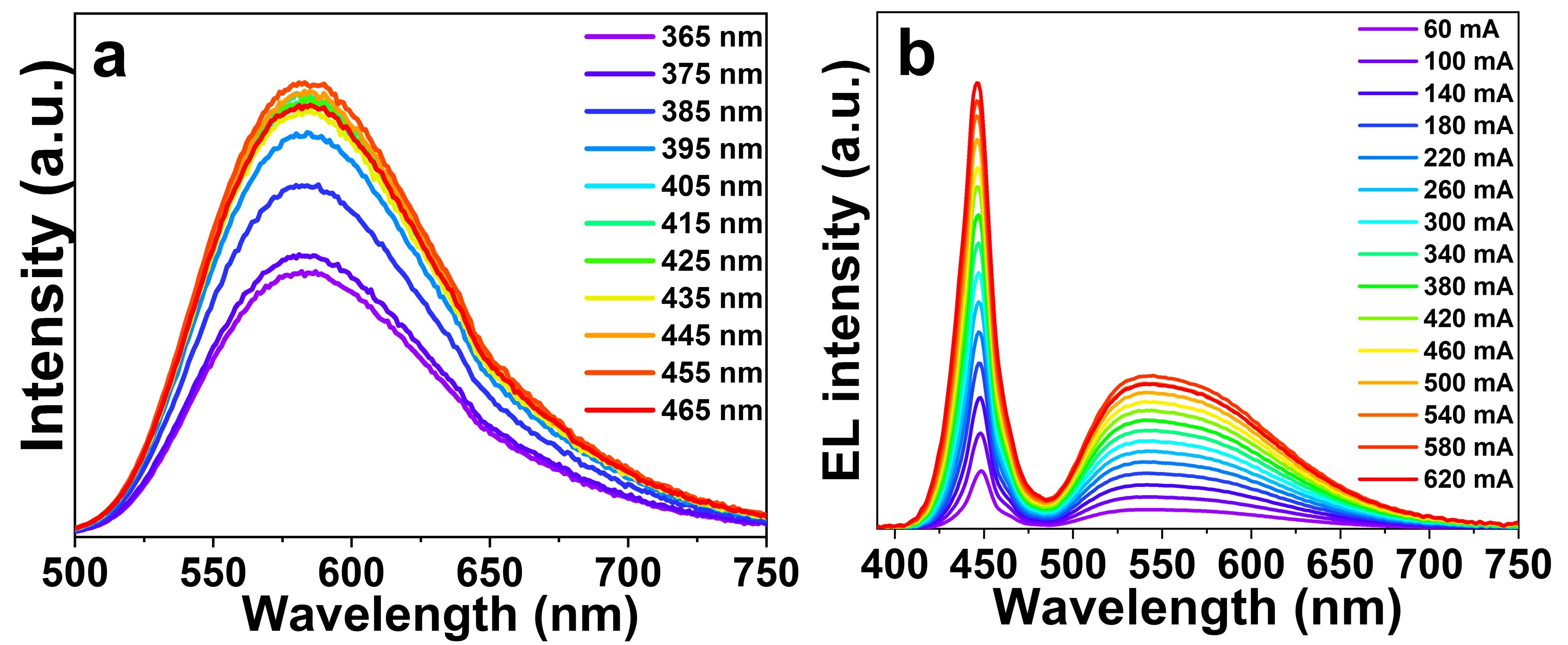


**Figure S2** (a) Emission spectra of the Li_2_SrSiO_4_:3%Eu^2+^,0.5%Cs^+^ phosphors excited by different wavelength; (b) EL spectra of the commercially used YAG:Ce^3+^ as a function of working current in the range of 60-620 mA.


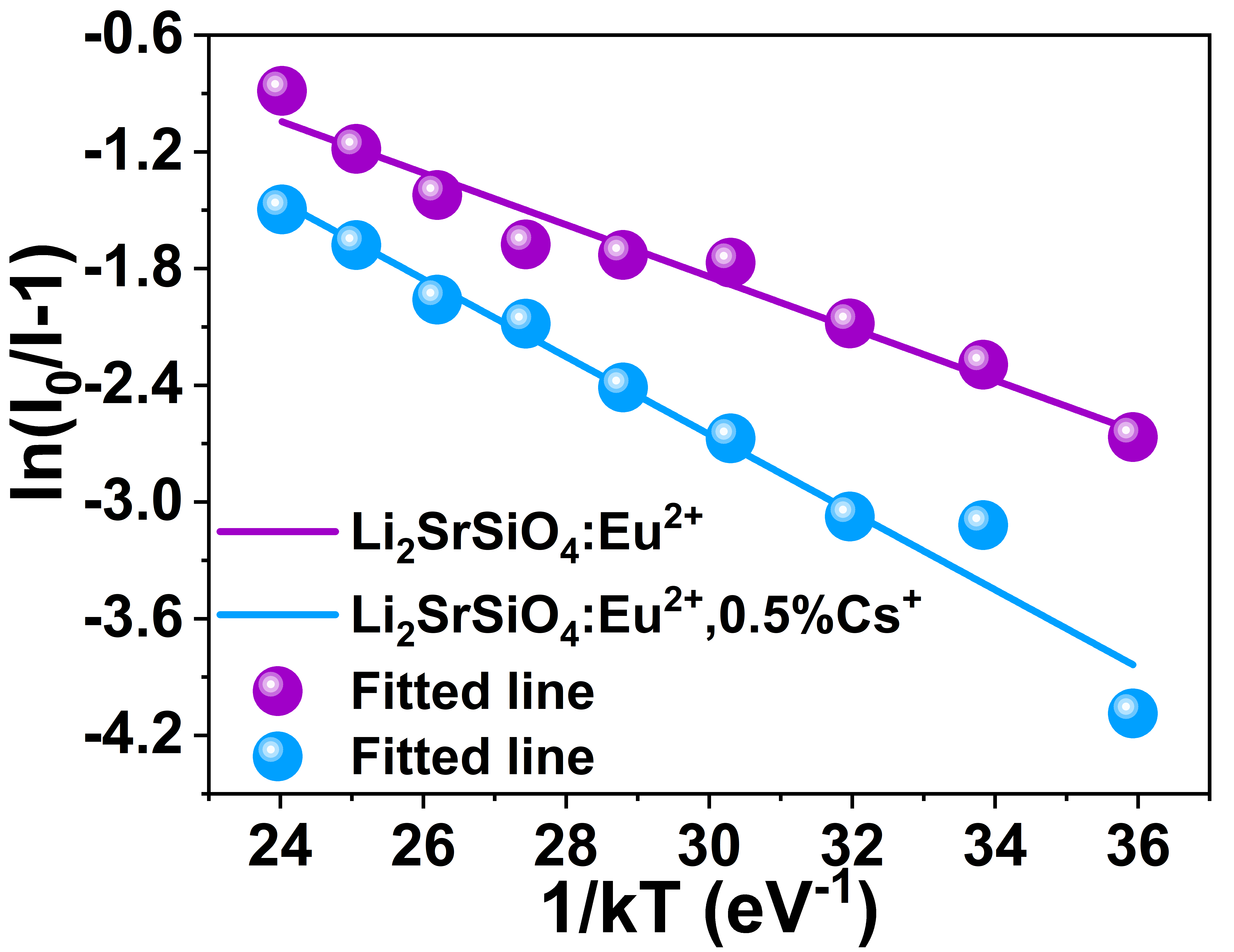


**Figure S3** Plots of the ln(I_0_/I-1) *vs*. 1/kT for the Li_2_SrSiO_4_:3%Eu^2+^ and Li_2_SrSiO_4_:3%Eu^2+^,0.5%Cs^+^ phosphors


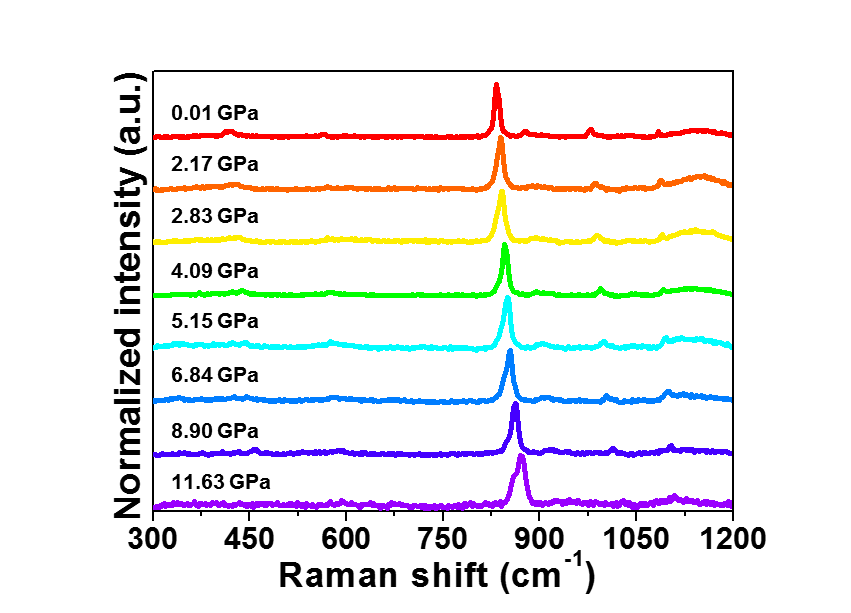


**Figure S4** Pressure-dependent Raman spectra of the Li_2_SrSiO_4_:3%Eu^2+^,0.5%Cs^+^ phosphors during the decompression process.


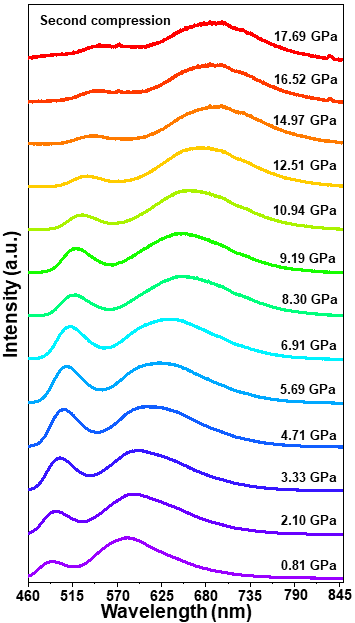


**Figure S5** Normalized emission spectra of the L_i2_SrSiO_4_:3%Eu^2+^,0.5%Cs^+^ phosphor during the second compression cycle under 450 nm excitation.


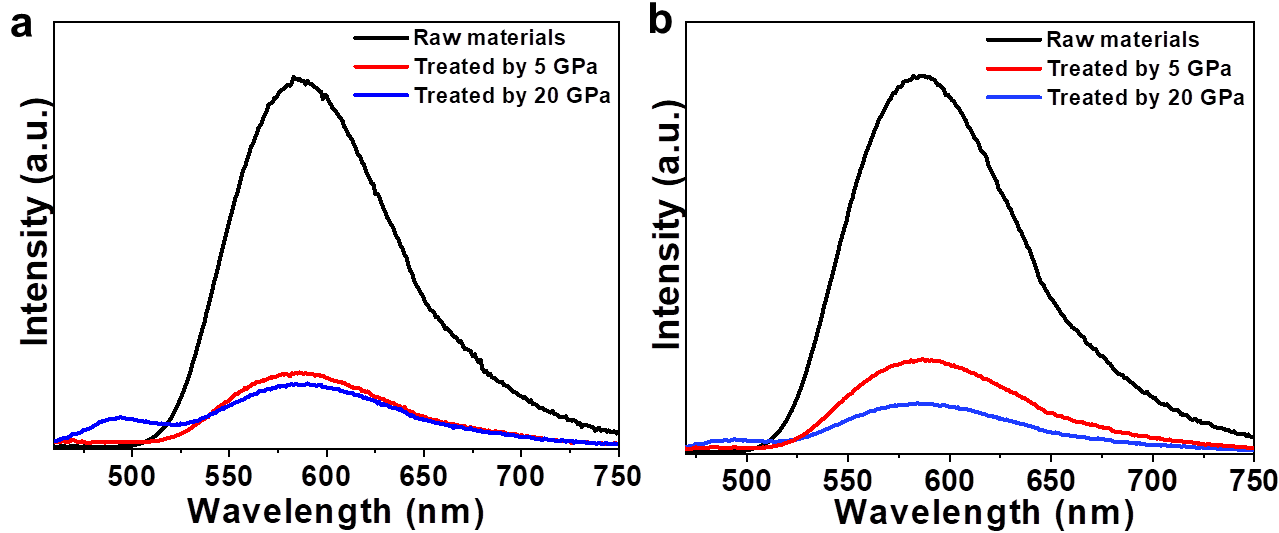


**Figure S6** Emission spectra of the designed materials, which were treated by different pressure conditions, excited at (a) 419 and (b) 450 nm.
